# Geschlechtsspezifische Aspekte bei Prädiabetes und Diabetes mellitus – klinische Empfehlungen (Update 2023)

**DOI:** 10.1007/s00508-023-02185-5

**Published:** 2023-04-20

**Authors:** Alexandra Kautzky-Willer, Michael Leutner, Heidemarie Abrahamian, Lisa Frühwald, Fritz Hoppichler, Monika Lechleitner, Jürgen Harreiter

**Affiliations:** 1 Gender Medicine Unit, Klinische Abteilung für Endokrinologie und Stoffwechsel, Universitätsklinik für Innere Medizin III, Medizinische Universität Wien, Währinger Gürtel 18–20, 1090 Wien, Österreich; 2 Internistisches Zentrum Baumgartner Höhe, Otto-Wagner-Spital, Wien, Österreich; 3 5. Medizinische Abteilung mit Endokrinologie, Rheumatologie und Akutgeriatrie, Klinik Ottakring, Wien, Österreich; 4 Interne Abteilung, Krankenhaus der Barmherzigen Brüder Salzburg, Salzburg, Österreich; 5 Avomed-Arbeitskreis für Vorsorgemedizin und Gesundheitsförderung in Tirol, Innsbruck, Österreich

**Keywords:** Geschlechterdifferenz, Diabetes, Kardiovaskluläre Erkrankungen, Diabeteskomplikationen, Prävention, Sex and Gender, Diabetes, Cardiovascular disease, Diabetes-related complications, Prevention

## Abstract

Metabolische Erkrankungen beeinflussen das Leben von Männern und Frauen in den verschiedenen Lebensabschnitten in unterschiedlicher und vielfältiger Weise und stellen eine große Herausforderung für das Gesundheitssystem dar. Die behandelnden Ärztinnen und Ärzte sind mit den unterschiedlichen Bedürfnissen von Männern und Frauen im klinischen Alltag konfrontiert. Geschlechtsspezifische Unterschiede beeinflussen die Pathophysiologie, das Screening und die Diagnose von Krankheiten, sowie Behandlungsstrategien und die Entwicklung von Komplikationen und die Mortalitätsraten. Veränderungen im Glukose- und Lipidstoffwechsel, die Regulation von Energiehaushalt und Körperfettverteilung sowie damit assoziierte kardiovaskuläre Erkrankungen werden stark von Steroid- und Sexualhormonen beeinflusst. Zusätzlich spielen Erziehung, Einkommen und psychosoziale Faktoren eine wichtige Rolle bei der Entstehung von Adipositas und Diabetes und müssen bei geschlechtsspezifischer Betrachtung mitberücksichtigt werden. Männer weisen im jüngeren Alter und bei niedrigerem BMI ein höheres Risiko für Typ 2 Diabetes auf als Frauen, die wiederum von einem starken Anstieg im Risiko für Diabetes-assoziierte kardiovaskuläre Erkrankungen nach der Menopause betroffen sind. Frauen dürften durch Diabetes auch etwas mehr Lebensjahre verlieren als Männer, wobei die höhere Mortalität hauptsächlich auf vaskuläre Komplikationen zurückgeführt werden kann. Bei Männern mit Diabetes scheint dafür der Mortalitätsanstieg durch Krebs gewichtiger als bei Frauen zu sein. Bei Frauen sind Prädiabetes und Diabetes meist mit mehr vaskulären Risikofaktoren assoziiert wie erhöhte Inflammationsparameter, prothrombotische Veränderungen und höherem Blutdruck. Sie weisen deshalb ein relativ höheres vaskuläres Risiko auf. Frauen sind öfter stark übergewichtig und weniger körperlich aktiv, obwohl sie sogar noch mehr als Männer von einem höheren Bewegungsausmaß in ihrer Gesundheit und Lebenserwartung profitieren dürften. In Gewichtsreduktionsprogrammen verlieren Männer häufig mehr Gewicht als Frauen. Frauen und Männern profitieren gleich gut von Präventionsprogrammen mit etwa 40 % Risikoreduktion für Typ 2 Diabetes nach 3 Jahren. Langzeitdaten konnten bisher eine Reduktion der allgemeinen und kardiovaskulären Mortalität nur bei Frauen zeigen. Frauen weisen öfter eine gestörte Glukosetoleranz, Männer hingegen erhöhte Nüchternblutzuckerspiegel auf. Eine Anamnese eines Gestationsdiabetes oder polyzystischen Ovarsyndroms (PCOS) sowie höhere Androgenspiegel, und erniedrigte Östrogenspiegel stellen bei Frauen, das Vorhandensein einer erektilen Dysfunktion oder erniedrigter Testosteronspiegel bei Männern, wichtige geschlechtsspezifische Diabetesrisikofaktoren dar. Viele Studien zeigen des Weiteren, dass Frauen in der Therapie weniger oft die Zielwerte für HbA_1c_, LDL-Cholesterin oder Blutdruck erreichen, wobei die Ursachen unklar sind. Generell sollen in der medikamentösen Behandlung geschlechtsspezifische Unterschiede in der Wirkung, Pharmakokinetik und in den Nebenwirkungen mehr Beachtung finden.

## Abstract (en)

Metabolic diseases dramatically affect the life of men and women from infancy up to old age in different and manifold ways and are a major challenge for the healthcare system. The treating physicians are confronted with the different needs of women and men in the clinical routine. Gender-specific differences affect pathophysiology, screening, diagnostic and treatment strategies of diseases as well as the development of complications and mortality rates. Impairments in glucose and lipid metabolism, regulation of energy balance and body fat distribution and therefore the associated cardiovascular diseases, are greatly influenced by steroidal and sex hormones. Furthermore, education, income and psychosocial factors play an important role in the development of obesity and diabetes differently in men and women. Males appear to be at greater risk of diabetes at a younger age and at a lower body mass index (BMI) compared to women but women feature a dramatic increase in the risk of diabetes-associated cardiovascular diseases after the menopause. The estimated future years of life lost owing to diabetes is somewhat higher in women than men, with a higher increase in vascular complications in women but a higher increase of cancer deaths in men. In women prediabetes or diabetes are more distinctly associated with a higher number of vascular risk factors, such as inflammatory parameters, unfavourable changes in coagulation and higher blood pressure. Women with prediabetes and diabetes have a much higher relative risk for vascular diseases. Women are more often morbidly obese and less physically active but may have an even greater benefit in health and life expectation from increased physical activity than men. In weight loss studies men often showed a higher weight loss than women; however, diabetes prevention is similarly effective in men and women with prediabetes with a risk reduction of nearly 40%. Nevertheless, a long-term reduction in all cause and cardiovascular mortality was so far only observed in women. Men predominantly feature increased fasting blood glucose levels, women often show impaired glucose tolerance. A history of gestational diabetes or polycystic ovary syndrome (PCOS) as well as increased androgen levels and decreased estrogen levels in women and the presence of erectile dysfunction or decreased testosterone levels in men are important sex-specific risk factors for the development of diabetes. Many studies showed that women with diabetes reach their target values for HbA_1c_, blood pressure and low-density lipoprotein (LDL)-cholesterol less often than their male counterparts, although the reasons are unclear. Furthermore, sex differences in the effects, pharmacokinetics and side effects of pharmacological treatment should be taken more into consideration.

## Grundsatzstatement

Das Geschlecht beeinflusst das Gesundheitsbewusstsein und -verhalten in unterschiedlicher Weise. Neben den biologischen (genetisch und hormonell bedingten) geschlechtsspezifischen Unterschieden sind auch jene als Folge des Einflusses von Gesellschaft, Kultur, Geschlechterrollen und psychosozialen Faktoren zu bewerten und in der Kommunikation, bei der Prävention, der Diagnose und Therapie des Diabetes zu berücksichtigen [1].

## Epidemiologie

In Österreich liegt die Lebenserwartung der Frauen 2020 bei 83,7 Jahren und bei Männern bei 78,9 Jahren. Die Diabetesprävalenz von 20- bis 79-jährigen Frauen lag 2021 bei geschätzt 10,2 %, was geringfügig niedriger ist als jene der Männer mit 10,8 %. Weltweit haben etwa 17,7 Mio. mehr Männer Diabetes als Frauen [2]. Diabetes ist bei Frauen einer der stärksten Risikofaktoren für Herz-Kreislauf-Erkrankungen, die insgesamt 38,7 % der Todesursachen der Frauen und 32,5 % der Männer in Österreich 2020 ausmachten [3, 4]. In einer gepoolten Analyse prospektiver Studien war die Mortalitätsrate bei Männern mit Diabetes krebsbedingt und bei Frauen mit Diabetes vaskulär bedingt besonders stark erhöht im Vergleich zu Gruppen ohne Diabetes gleichen Geschlechts [5].

Ein niedriger Sozialstatus und schlechte Bildung sind mit einem höheren Risiko für Diabetes verbunden. Auswertungen aus der Gesundheitsbefragung in Österreich 2007 ergaben außerdem, dass der inverse Zusammenhang zwischen Bildungsgrad und Auftreten von Übergewicht und Diabetes bei Frauen stärker ist als bei Männern [6].

## Klassifikation und Diagnose

Die Prävalenz von Diabetes mellitus Typ 1 und Typ 2 ist bei beiden Geschlechtern annähernd gleich mit einem leichten männlichen Überhang. Lediglich im Kindesalter sind bis zur Pubertät mehr Mädchen als Buben von Typ 2 Diabetes (T2DM) betroffen. Generell ist T2DM im Kindesalter in Österreich jedoch selten. Ein zunehmendes Problem in der Jugend ist die steigende Adipositasprävalenz und damit verbunden auch ein erhöhtes Risiko für Störungen des Glukosestoffwechsels. Eine rezente Studie zeigte, dass im Kindesalter und in der Jugend mehr Buben als Mädchen in Ländern mit hohen Einkommen von Adipositas betroffen sind [7]. Interessanterweise wurde in einer rezenten Untersuchung eine höhere T2DM-Prävalenz sowie NAFLD (nicht-alkoholische Fettlebererkrankung) bei Mädchen beobachtet [8]. Im Erwachsenenalter scheinen Männer häufiger im mittleren Lebensalter und bei niedrigerem Body Mass Index (BMI) als Frauen einen Diabetes zu manifestieren [9]. Europäische populationsbezogene Verlaufsbeobachtungen weisen auf eine zwischen den Geschlechtern vergleichbare (Bruneck Study) oder für Männer höhere (KORA S4/F4 Cohort Study) Diabetesinzidenz hin [9].

Bezüglich des Stadiums „Prädiabetes“ liegt bei Frauen häufiger das Stadium der gestörten Glukosetoleranz vor, während bei Männern die erhöhte Nüchternglukose überwiegt [9, 10]. Aufgrund dessen ist die Durchführung eines OGTTs bei der Diagnostik von Glukosestoffwechselstörungen vor allem bei Frauen besonders wichtig. Bei Frauen nach Gestationsdiabetes (GDM) zeigten Studien, dass sogar bei einem Großteil eine Glukosetoleranzstörung nur anhand erhöhter 2‑h-Blutzuckerwerte im oralen Glukosetoleranztest erkannt wurde [11]. Zur höheren Rate an gestörter Glukosetoleranz (IGT) von Frauen könnten deren geringere Körpergröße und fettfreie Masse sowie eine verlängerte Darmglukoseaufnahme beitragen [12]. Eine ausgeprägtere Insulinsekretionsstörung in der frühen Phase sowie eine gesteigerte Glukoseausschüttung in der Leber kombiniert mit einer geringeren Insulinsensitivität tragen zu bei Männern häufiger vorkommenden erhöhten Nüchternglukosewerten bei [9].

## Metabolisches Syndrom

Adipositas betrifft häufiger Frauen als Männer, während das Vollbild des metabolischen Syndroms je nach Definition bei beiden Geschlechtern unterschiedlich häufig beschrieben wird. Während die IDF-Kriterien annähernd gleich viele Männer und Frauen mit einem metabolischen Syndrom klassifizieren, sind durch die NCEP-ATP III oder WHO-Kriterien mehr Männer als Frauen betroffen [9]. Bei allen Definitionen sind dabei die geschlechtsspezifischen Grenzwerte für HDL-Cholesterin und den Bauchumfang bzw. die „Waist-to-Hip-Ratio“ zu beachten. Unabhängig vom BMI ist ein Bauchumfang über 102 cm bei Männern und über 88 cm bei Frauen mit einer Zunahme des Mortalitätsrisikos um ungefähr 30 % bei den beiden Geschlechtern verbunden [13]. Bei Frauen ist der Bauchumfang ein besserer Prädiktor für Diabetes als der BMI.

## Sexualität

Die Ätiologie von Sexualfunktionsstörungen ist multikausal und betrifft vor allem vaskuläre, neurogene, hormonelle und psychische Komponenten, deren Zusammenwirken für eine intakte Sexualfunktion Voraussetzung ist. Vaskuläre Funktionsstörungen wie endotheliale Dysfunktion bis hin zur manifesten Atherosklerose sind bei Männern als Ursache einer erektilen Dysfunktion (ED) sehr gut dokumentiert. Männer mit Diabetes mellitus haben ein erhöhtes Risiko für erektile Dysfunktion [9]. Da in der Regel sowohl eine endotheliale Dysfunktion als auch Atherosklerose bei Gefäßen mit kleinem Lumen früher klinisch manifest werden als bei Gefäßen mit größerem Lumen, wie z. B. Koronargefäßen, wird bei Vorliegen einer ED ein umfassendes Screening auf Makroangiopathie, insbesondere auf koronare Herzkrankheit, empfohlen [14]. Die ED stellt mit hoher Wahrscheinlichkeit einen unabhängigen Marker für das kardiovaskuläre Risiko dar [9, 15]. Auch die diabetische Polyneuropathie kann zu Sexualfunktionsstörungen bei Männern mit Diabetes beitragen, sowohl als Manifestation im peripheren als auch im autonomen Nervensystem. Oxidativer Stress, gestörter Sorbitol-Stoffwechsel und Mangel an Nervenwachstumsfaktoren spielen unter anderem eine wichtige Rolle [16]. Als hormonelle Ursache für Sexualfunktionsstörungen steht der Testosteronmangel mit entsprechenden Auswirkungen auf Libido und Erregung beim männlichen Geschlecht an erster Stelle. Viszerale Adipositas im Rahmen von Diabetes und metabolischem Syndrom sowie die Gesamtfettmasse sind wesentliche Faktoren eines Testosteronmangels bei diesen Patienten [9, 15].

Bei Frauen mit Diabetes sind die kausalen pathophysiologischen Zusammenhänge ähnlich wie bei Männern mit Diabetes gelagert: endotheliale Dysfunktion und Atherosklerose, Störungen der genitalen Innervation und Veränderungen im Hormon- und Neurotransmittermuster, sowie eine ED der Klitoris wurden beschrieben. Darüber hinaus sind bei Frauen auch noch mechanische Irritationen der Schleimhäute durch unmittelbare Auswirkungen der Glykämie mit Beeinträchtigungen der Lubrikation zu berücksichtigen [17]. Die Prävalenz weiblicher sexueller Dysfunktion ist bei Frauen mit Diabetes erhöht, wird aber meist im Diabetesmanagement nicht berücksichtigt und dementsprechend nicht diagnostiziert oder behandelt [18]. Verminderte sexuelle Appetenz („hypoactive sexual desire disorder“ [HSDD] oder „female sexual interest/arousal disorder“ – Nomenklatur nicht einheitlich) ist das am häufigsten geäußerte Problem.

Ein gering höherer, möglicherweise klinisch bedeutsamer Benefit, wurde im Vergleich zu Placebo unter Flibanserin beschrieben, jedoch ist zur optimalen Versorgung eine multidisziplinäre Vorgehensweise erforderlich [18, 19]. Bei Verschreibung von Flibanserin muss eine Aufklärung zu möglichen Nebenwirkungen wie schwere Hypotonie, Schwindel, Synkope und Somnolenz v. a. in Zusammenhang mit Alkoholeinnahme, erfolgen [18]. Studien zur Wirksamkeit von Flibanserin bei Frauen mit Diabetes liegen bisher nicht vor, weshalb eine Verschreibung vom behandelnden Arzt/von der behandelnden Ärztin in Absprache mit der Patientin hinsichtlich des geringen zu erwartenden Effekts und der möglichen schweren Nebenwirkungen und Kosten abzuwägen ist [18]. Andere Medikamente zur Verbesserung der weiblichen Sexualfunktionsstörung wie Testosteron oder Bupropion werden „off label“ eingesetzt und zeigen lediglich moderate Effekte.

Sexuelle Dysfunktion als Nebenwirkung von Medikamenten spielt sowohl bei Männern als auch bei Frauen mit Diabetes eine Rolle. Dabei stehen vor allem bestimmte Antidepressiva mit Auswirkungen auf den Prolaktinstoffwechsel und reduzierter Bildung von Sexualhormonen im Fokus des Interesses. Da bei Patient:innen mit Diabetes bei ca. 18 % eine behandlungsbedürftige Depression vorliegt, bei Frauen mit Diabetes häufiger als bei Männern mit Diabetes, ist das jeweilige Antidepressivum sorgfältig auszuwählen, um Nebenwirkungen abzuwenden [20].

Als Screeningmethode empfiehlt sich bei Patient:innen mit Diabetes zumindest einmal jährlich das sorgfältige Erheben einer Sexualanamnese. Für die ergänzende Diagnostik bieten sich validierte Fragebögen wie International Index of Erectile Function-5-Score (IIEF5) für Männer [21] und Female Sexual Function Index (FSFI) für Frauen an [22]. Beide enthalten Fragen zu den wichtigen Dimensionen der Sexualität wie Libido, Erektion, Lubrikation, Orgasmus, Befriedigung und Schmerz. Bei Vorliegen einer Sexualfunktionsstörung sollte ein kompletter Status der Sexualhormone erhoben werden.

Die Therapie der Sexualfunktionsstörung orientiert sich an den Ursachen, jedoch stehen generell Lebensstilinterventionen und Tabakabstinenz im Vordergrund des Therapieansatzes. Bei vaskulärer Ursache empfiehlt sich die kontinuierliche Therapie der Atherosklerose, bei neuropathischer Ursache ist die Blutzuckereinstellung eine wichtige Maßnahme, bei hormonellen Defiziten kann die Hormonsubstitution in Erwägung gezogen werden. Der Einsatz von PDE-5-Hemmern führt bei Männern zu einer Besserung der Erektion durch Vasodilatation mit gesteigertem Blutfluss durch erhöhte Bereitstellung von Stickstoffmonoxid (NO). Der Einsatz bei Frauen zeigt kontroverse Ergebnisse und wird nur bei SSRI-induzierter Sexualfunktionsstörung empfohlen [16].

Bei psychischer Ursache der Sexualfunktionsstörung kommt psychotherapeutischen Interventionen eine wesentliche Bedeutung zu.

## Lebensstil und Prävention

Lebensstilmaßnahmen können bei beiden Geschlechtern zu einer Reduktion der Hyperglykämie und Risikoreduktion für Diabetes beitragen [15]. Acarbose wirkte bei älteren normal- bis übergewichtigen Frauen ohne Hypertonie besser, Metformin vor allem bei jüngeren adipösen Männern mit erhöhten Nüchternblutzuckerwerten [11]. In einer Metaanalyse konnte gezeigt werden, dass eine Lebensstilintervention das Diabetesrisiko bei Männern und Frauen nach 3 Jahren gleichermaßen um etwa 40 % senkt [23]. Im Follow-up der Da-Qing-Studie nach 30 Jahren wurde eine signifikante Reduktion der kumulativen Inzidenz von kardiovaskulären Events um 30 % und der allgemeinen Mortalität um 41 % bei Frauen im Lebensstilinterventionsarm im Vergleich zu weiblichen Kontrollen ohne Lebensstilmaßnahmen gesenkt. Bei Männern konnte hinsichtlich kardiovaskulärer Events und der allgemeinen Mortalität eine nicht signifikante Reduktion um 20 % bzw. 15 % festgestellt werden[24], obwohl die Senkung der kumulativen Diabetesinzidenz bei Männern (39 %) und Frauen (38 %) vergleichbar war. Interessanterweise zeigte sich in der LOOK-AHEAD Studie, dass übergewichtige/adipöse Männer mit T2DM durch Lebensstilmaßnahmen innerhalb eines Zeitraums von 4 Jahren mehr Gewicht verloren haben [25]. In der rezent publizierten randomisiert kontrollierten T4DM Studie konnte gezeigt werden, dass eine Testosteronsupplementation mit 1000 mg Testosteron-undecanoate bei Männern mit niedrigen Testosteronwerten aber keinem pathologischen Hypogonadismus, IGT oder neudiagnostiziertem T2DM und erhöhtem Bauchumfang im Vergleich zu Placebo die Anzahl der Patienten mit T2DM nach 2 Jahren Behandlung signifikant um 41 % reduzieren konnte[26].

Bei Frauen scheinen bereits Prädiabetes und das metabolische Syndrom stärker als bei Männern mit erhöhten Inflammationsparametern, einer ungünstigeren Veränderung im Gerinnungssystem und höheren Blutdruckwerten einherzugehen [9, 27]. Dies bestätigt sich auch bei manifestem Diabetes und könnte zum besonders stark erhöhten kardiovaskulären Risiko bei Frauen beitragen [22, 28].

Des Weiteren wurde für Frauen bestätigt, dass anamnestisch erhobene reproduktive Faktoren (Parität, Zyklusunregelmäßigkeiten, Präeklampsie, frühe Menopause) sowie insbesondere die Anamnese eines früheren GDM mit dem Ausmaß der aktuellen Stoffwechselstörung eng assoziiert sind [15]. Frauen mit einem früheren GDM konvertierten bei vergleichbarer Studienausgangslage bezüglich Insulinresistenz, Körpergewicht und Glukosetoleranzstatus fast doppelt so häufig zu manifestem Diabetes wie jene ohne GDM in der Anamnese. Durch Lebensstilmaßnahmen und Metformin kann jedoch die Progression zu Typ 2 Diabetes signifikant um fast 40 % im Vergleich zur Kontrolle langfristig reduziert werden [29].

Diese Daten unterstützen bei Frauen die Wichtigkeit der gynäkologischen/geburtshilflichen Anamnese und die Notwendigkeit regelmäßiger, engmaschiger Nachuntersuchung nach GDM (s. auch Leitlinie Gestationsdiabetes). Bei Frauen ist in diesem Zusammenhang noch das polyzystische Ovarsyndrom, das ungefähr 10 % aller Frauen betrifft und durch erhöhte Androgenspiegel, Zyklusunregelmäßigkeiten und/oder polyzystisches Ovar charakterisiert ist und häufig von Insulinresistenz begleitet wird [30], als geschlechtsspezifischer Risikofaktor für einen Prädiabetes oder Diabetes hervorzuheben, der unbedingt bezüglich einer Glukosestoffwechselstörung abgeklärt werden soll. Demgegenüber stehen Männer mit niedrigem Testosteronspiegel, die ebenso ein erhöhtes Risiko für Diabetes aufweisen und auch in Screeningmaßnahmen einbezogen werden sollten (siehe auch Leitlinie Diabetes mellitus – Definition, Klassifikation, Diagnose, Screening und Prävention) [15].

## Krankheitsbewältigung

Psychosoziale Faktoren beeinflussen die Krankheitsbewältigung und Coping-Strategien bei Männern und Frauen unterschiedlich [31]. Frauen beschäftigen sich generell intensiver mit ihrer Erkrankung und sind besser über Diabetes informiert als Männer. Zusätzlich spielen bei Frauen emotionale Faktoren und der Bezug zum behandelnden Arzt/der behandelnden Ärztin eine größere Rolle. Männer wiederum profitieren besonders von strukturierten evidenzbasierten Diabetes-Management-Programmen. Diabetes verschlechtert die gesundheitsbezogene Lebensqualität bei Frauen stärker als bei Männern [32].

## Orale Antidiabetika, GLP-1-Analoga und Insulin

Frauen erreichen unter Therapie weniger häufig die HbA_1c_-Ziele trotz höherem Risiko für Hypoglykämien [33]. Unter einer Kombinationstherapie mit Metformin- und Sulfonylharnstoffen wurde eine stärkere HbA_1c_ Senkung bei Männern berichtet [34, 35]. Im Nebenwirkungsprofil einiger Medikamente gibt es geschlechtsspezifische Unterschiede (Tab. 1). So weisen postmenopausale Frauen unter Glitazontherapie, trotz eines möglicherweise effektiveren blutzuckersenkenden Effekts im Vergleich zu Männern bei Adipositas [34], häufiger Knochenbrüche auf. Die Ursache für diesen Geschlechtsdimorphismus ist bisher unklar. In der Substanzklasse der SGLT2-Hemmer sind häufiger Ketoazidosen, insbesondere aber Harnwegsinfekte und Genitalinfektionen, v. a. Pilzinfektionen, bei Frauen beschrieben (Risiko für Frauen bereits vor Beginn einer SGLT2-Inhibitortherapie 3‑fach höher als bei Männern, unter Therapie bei beiden Geschlechtern erhöht), v. a. wenn schon früher rezidivierend Infektionen im Urogenitalbereich aufgetreten sind.

**Table 1:** 

Substanz	Bemerkungen
Glitazone	Höheres Knochenfrakturrisiko bei postmenopausalen Frauen
SGLT2-Hemmer	Harnwegsinfekt, Vulvovaginitis und Balanitis und assoziierte genitale Infekte, mit größerem Risiko für Frauen Höheres Risiko für Ketoazidose bei Frauen Vergleichbarer Effekt auf kardiovaskuläre Mortalitätsreduktion und Reduktion der Hospitalisierungen wegen Herzinsuffizienz
GLP1-Rezeptoragonisten	Höhere kardiovaskuläre Risikoreduktion bei Frauen im Vgl. zu Männern, höhere Häufigkeit von GIT-Beschwerden bei Frauen
Insulin	Frauen: Hypoglykämierisiko nach Insulingabe höher, besonders stark erhöht bei schlanken Frauen Männer: Bei normalgewichtigen ebenso höheres Hypoglykämierisiko als bei stark übergewichtigen und adipösen Männern
Lipidsenkende Therapie	Atheromvolumenreduktion bei Frauen mit Rosuvastatin und Evolocumabtherapie höher Lipidsenkender Effekt bei Frauen unter Fenofibrat größer Statine: Häufiger Nebenwirkungen bei älteren Frauen mit niedrigem Körpergewicht
Thrombozytenaggregationshemmer	Primärprävention: Risikoreduktion von kardiovaskulären Ereignissen um 11 % bei Männern, nicht bei Frauen Allgemein in der Primärprävention keine Empfehlung für Patient:innen mit moderatem kardiovaskulären Risiko oder hohem Alter
β‑Blocker	Erhöhte Blutdruck- und Herzfrequenzreduktion bei Sport betreibenden Frauen
Kalziumkanalblocker	Stärkere Blutdrucksenkung bei Frauen Erhöhte Ödeminzidenz bei Frauen
ACE-Inhibitoren	Erhöhte Reizhusteninzidenz bei Frauen

*SGLT2* Sodium-Glucose Co-Transporter 2, *ACE* Angiotensin Converting Enzyme

Unter Basalinsulintherapieeinsatz wurden besonders bei nur leicht übergewichtigen Frauen häufiger schwere Hypoglykämien beschrieben [33], vermutlich aufgrund höherer Insulindosen bezogen auf das Körpergewicht. Dies trifft aber auch für normalgewichtige und leicht übergewichtige Männer zu. Bei Exenatid werden häufiger gastrointestinale Beschwerden sowie tendenziell höherer Gewichtsverlust und Reduktion von Nüchternglukose und Blutdruck bei Frauen beschrieben. In allen kardiovaskulären Outcomestudien zu SGLT2-Hemmern oder GLP-1RA waren insgesamt weniger Frauen eingeschlossen, die auch im Beobachtungsverlauf weniger kardiovaskuläre Ereignisse zeigten. Die kardiovaskuläre Risikoreduktion war bei Frauen mit Diabetes in allen diesen kardiovaskulären Outcome-Studien nicht signifikant, obwohl keine Interaktion mit dem Geschlecht gefunden und positive Trends bestätigt wurden [36]. Diese Unterschiede zwischen Männern und Frauen sind wohl durch die geringere Anzahl an Studienteilnehmerinnen sowie der generell niedrigeren Rate an kardiovaskulären Ereignissen im Beobachtungszeitraum bei den Frauen und der daraus resultierenden niedrigeren statistischen Power bedingt. Jedenfalls bestätigte eine gepoolte Analyse von Outcome-Studien für beide Substanzklassen eine signifikante Senkung des kardiovaskulären Risikos (primäre kardiovask. Outcomes) bei Männern wie auch bei Frauen [37]. Auch in einer systematischen amerikanischen Übersichtsarbeit erwies sich der positive Effekt auf die kardiovaskuläre Mortalität und Hospitalisierung wegen Herzinsuffizienz bei Männern und Frauen als vergleichbar [38]. Trotzdem wurde gezeigt, dass SGLT2-Hemmer bei Frauen mit T2DM selbst bei bekannter KHK, Nieren- oder Herzinsuffizienz, ebenso wie generell bei sozial benachteiligten Menschen seltener angewendet wurden [39]. Im Vergleich zu Sulfonylharnstoffen waren sowohl DPP4-Hemmer, GLP1-Rezeptoragonisten als auch SGLT2-Hemmer mit weniger kardiovaskulären Ereignissen (Myokardinfarkt/instabile Angina, Schlaganfall oder Herzinsuffizienz) bei beiden Geschlechtern assoziiert [40], wobei bei Frauen durch GLP1-RA signifikant bessere kardiovaskuläre Effekte erzielt wurden als bei Männern.

## Multifaktorielles Risikomanagement

Frauen mit Diabetes erreichen weniger häufig die leitlinienkonformen Therapieziele für HbA_1c_, Blutdruck und/oder Lipide [36, 41]. Das könnte auf Unterschiede in der Rate an Komorbiditäten, an Krankheitssymptomen, in der ärztlichen Einschätzung der Gefährdung der Patientinnen und Patienten bzw. dem ärztlichen Kommunikations- und Verordnungsmodus, in der Therapieadhärenz oder aber auch in der allgemein höheren Nebenwirkungsrate in der Pharmakotherapie bei Frauen zurückgeführt werden. Allerdings reagieren Frauen auf bestimmte kardiovaskuläre Risikomarker sogar empfindlicher im Risikoanstieg für Komplikationen als Männer. Frauen haben eine höhere Salzsensitivität und reagieren auf Salzzufuhr mit einem deutlicheren Blutdruckanstieg, profitieren aber auch stärker bei Salzrestriktion. Ebenso führen hohe Serumtriglyzeride und niedrige HDL-Cholesterinwerte bei Frauen zu einem höheren Anstieg im kardiovaskulären Risiko. Rauchen ist bei Frauen mit einem um 25 % höheren Risiko für Myokardinfarkte verbunden als bei Männern und sollte bei beiden Geschlechtern bei Diabetes unbedingt vermieden werden (siehe Leitlinie Diabetes und Rauchen, Alkohol). Unterschätzte kardiovaskuläre Risikofaktoren sind ein der Gestationsdiabetes, das PCOS und eine Schwangerschaftshypertonie oder Präeklampsie, die bei Frauen mit Diabetes öfter auftreten [4]. Junge Frauen mit Zustand nach Gestationsdiabetes haben ein 2‑fach höheres Risiko für kardiovaskuläre Events [42].

Bei Frauen steigt das Risiko für Adipositas, Dyslipidämie, Hypertonie und Glukosestoffwechselstörungen nach der Menopause deutlich an, weswegen ein regelmäßiges Monitoring dieser Risikoparameter in dieser Phase besonders wichtig ist [4, 43].

Besorgniserregend ist, dass insbesondere Hochrisikopatientinnen mit KHK eine schlechtere Kontrolle modifizierbarer kardiovaskulärer Risikofaktoren aufweisen und weniger häufig eine intensive lipidsenkende Therapie erhalten als Männer mit KHK [10, 36, 44]. Eine rezente systematische Übersichtsarbeit mit randomisiert kontrollierten kardiovaskulären Outcomestudien bei T2DM zeigte deutlich ein schlechteres kardiovaskuläres Risikomanagement mit höheren systolischen Blutdruckwerten, höherem LDL-Cholesterin und höherem HbA_1c_ bei den Frauen [45]. Frauen erhielten auch weniger häufig Statine, Betablocker oder Acetylsalicylsäure [45].

Statine wirken bei Frauen und Männern annähernd gleich [46]. In einer rezenten und bisher größten Metaanalyse konnte für beide Geschlechter ein vergleichbarer Benefit hinsichtlich vaskulärer und nichtvaskulärer Outcomes durch Atorvastatin und Rosuvastatin in der primären und sekundären Prävention beobachtet werden [47]. In der SATURN Studie konnte unter Hochdosis –Rosuvastatintherapie eine signifikant höhere prozentuelle Atheromvolumenreduktion bei Frauen im Vergleich zu Männern beobachtet werden [48]. Auch unter Evolocumabtherapie wurden ähnliche Resultate berichtet [49]. Unter einer Fenofibrattherapie im Vergleich zu Plazebo war der lipidsenkende Effekt bei Frauen mit T2DM ausgeprägter als bei Männern und mit einer – wenn auch nicht signifikant unterschiedlichen – höheren Reduktion kardiovaskulärer Events verbunden [50]. Bei den Antihypertensiva ist zu berücksichtigen, dass Frauen für die Auslösung von Arrhythmien durch QT-verlängernde Substanzen empfindlicher sind und bei Betablockern oft niedrigere Dosen als Männer benötigen. ACE-Hemmer scheinen bei Frauen die kardiovaskuläre Mortalität weniger stark zu senken, dafür aber eine Nephropathieentwicklung stärker zu verzögern, während AT-Rezeptor-Antagonisten bei Frauen besser wirksam sein könnten [51].

## Thrombozytenaggregationshemmer

Eine Therapie mit ASS ist bei Frauen mit einer geringeren antithrombotischen Wirkung und zudem auch mit einem höheren Blutungsrisiko assoziiert. ASS reduziert in der Primärprävention bei Frauen im Gegensatz zu Männern nicht das Myokardinfarktrisiko, wohl aber das Risiko für ischämische Insulte [52]. Eine rezente systematische Übersichtsarbeit zeigte bei Männern in der Primärprävention kardiovaskulärer Ereignisse eine signifikante relative Risikoreduktion von kardiovaskulären Ereignissen (MACE) mit einer Reduktion um 11 %, wohingegen bei Frauen der Effekt nicht signifikant war [53].

Viele Studien zeigen, dass Frauen mit kardiovaskulärem Risiko seltener ASS erhalten als Männer, obwohl für Frauen mit Diabetes in der Sekundärprävention ebenso wie für Männer ASS empfohlen wird (75–325 mg/Tag, Evidenzlevel A). In der Primärprävention sind die Daten weniger klar. Für Männer und Frauen mit Diabetes kann zwischen 50 und 70 Jahren auf individueller Basis und nach entsprechender Aufklärung und Nutzen-Risiko Abwägung eine Therapie mit ASS (75–162 mg/Tag) überlegt werden (Evidenzlevel B–C) [54, 55]. In der ASCEND Studie konnte zwar eine Reduktion von vaskulären Ereignissen unter ASS 100 mg bei Menschen mit Diabetes mellitus in der Primärprävention festgestellt werden, jedoch war auch die Ereignisrate für Blutungen um 29 % erhöht. Geschlechtsspezifische Unterschiede wurden weder bei Risikoreduktion vaskulärer Ereignisse noch im Blutungsrisiko festgestellt [56].

Aufgrund des erhöhten Blutungsrisiko und des geringen Nettobenefits in rezent publizierten RCTs und Metaanalysen wird in internationalen Leitlinien die Verwendung von ASS in der Primärprävention, vor allem in höherem Alter (> 70 Jahre) und bei moderatem Risiko für kardiovaskuläre Ereignisse (auch bei Diabetes mellitus) nicht empfohlen [57]. Bei Hochrisikopatienten kann ASS in Erwägung gezogen werden.

## Komplikationen und Komorbiditäten

### Makrovaskuläre Komplikationen

Während bei Männern mit und ohne Diabetes mellitus die kardiovaskuläre Mortalität im letzten Jahrzehnt abnahm, bleibt die Rate bei Frauen mit Diabetes unverändert hoch oder steigt sogar tendenziell an [9, 58]. Frauen mit Diabetes mellitus haben vor allem bei Diagnosestellung häufiger eine fortgeschrittene Atherosklerose verglichen mit Männern [34]. Ein höheres Risiko für Tod durch KHK ist bei Frauen mit Diabetes bekannt (Tab. 2). Neuere Studien konnten zeigen, dass diese geschlechtsspezifischen Unterschiede hinsichtlich kardiovaskulärer Mortalität aber auch der Entwicklung einer Herzinsuffizienz bei Diabetes mellitus weiterhin bestehen und in jüngeren Altersgruppen sogar noch deutlich ausgeprägter sind [59–61]. Daher scheinen auch Forderungen nach intensiveren Bemühungen für Screeningmaßnahmen für Glukosestoffwechselstörungen und anderen kardiovaskulären Risikofaktoren, sowie der frühzeitigen Etablierung prophylaktischer Maßnahmen gerechtfertigt [61].

**Table 2:** 

Parameter	Anmerkungen
Komorbiditäten	Frauen: allgemein höhere Belastung
Körperliche und kognitive Einschränkung/geriatrische Aspekte	Frauen: körperliche und kognitive Limitationen, Depressionen und Stürze häufiger
Depression/Angst	Frauen: höhere Depressions- und Angstprävalenz bei Diabetes Männer: stärkerer Zusammenhang zwischen Diabetes und Depression
Schwangerschaftskomplikationen (Frauen mit GDM oder Typ‑2-Diabetes mellitus)	Siehe Leitlinie Gestationsdiabetes und Gravidität bei vorbestehendem Diabetes
Nephropathie	Männer: schnellere Progression Frauen: höheres Risiko einer Proteinurie und Nierenerkrankung
Retinopathie	Männer mit Typ-1-Diabetes mellitus: bei Manifestation nach dem 15. Lebensjahr stärkeres Risiko für Entwicklung einer proliferativen Retinopathie und Nierenversagen, Männer: höheres Risiko bei T2DM
Sensorische Neuropathie	Männer: höheres Risiko bei T2DM
Diabetischer Fuß	Männer: höheres Risiko für Fußulzerationen, peripher vaskulären Komplikationen und Neuropathie
Koronare Herzkrankheit	Frauen: 40 % höheres relatives Risiko für koronare Herzkrankheit, letale und nicht letale Events
Herzinsuffizienz	Frauen: höheres relatives Risiko
Schlaganfall	Frauen: 27 % höheres relatives Risiko für Schlaganfall, letale und nicht letale Events

*GDM* Gestationsdiabetes

Nach einem Myokardinfarkt haben Frauen eine schlechtere Prognose. Dabei dürfte vor allem der fehlende protektive Effekt der weiblichen Geschlechtshormone eine Schlüsselrolle einnehmen. Die Symptome eines akuten Koronarsyndroms sind bei Frauen oft komplex und untypisch mit stärkerer vegetativer Ausprägung (Müdigkeit, Abgeschlagenheit, Übelkeit, Hals‑, Kiefer- oder Rückenschmerzen etc.) und werden deshalb häufiger fehlinterpretiert; Hypertonie ist besonders bei Frauen mit Diabetes ein wichtiger Risikofaktor für KHK, aber auch für Herzinsuffizienz. Daraus folgt, dass die Blutdruckkontrolle bei Frauen strikt verfolgt werden muss. Eine Metaanalyse stellte bei Frauen und Männer mit T1DM oder T2DM ein höheres Risiko für Herzinsuffizienz im Vergleich zu gesunden Frauen und Männern fest [62]. Im direkten Vergleich zu Männern haben aber Frauen mit T1DM oder T2DM das höhere relative Risiko (T1DM 47 %, T2DM 9 %). Auch das Risiko für eine Hospitalisierung wegen Herzinsuffizienz ist bei Frauen mit T2DM höher [45, 63]. In einer niederländischen Observationsstudie wurde eine 25 % höhere Inzidenzrate für den primären Endpunkt (Mortalität oder Hospitalisierung wegen Herzinsuffizienz) bei Männern mit bekannter chronischer Herzinsuffizienz beobachtet. Komorbiditäten wie Diabetes mellitus, Niereninsuffizienz oder chronische Lungenerkrankungen zeigten jedoch bei Frauen mit bekannter chronischer Herzinsuffizienz stärkere Assoziationen mit dem primären Endpunkt [64].

Frauen leiden öfter unter einer diastolischen, Männer öfter unter einer systolischen Dysfunktion. Frauen leiden auch wesentlich häufiger an einer HFpEF [4, 65]. Empagliflozin konnte auch in dieser Gruppe in der EMPEROR-Preserved-Studie den primären Endpunkt, eine Kombination von kardiovaskulärem Tod und Hospitalisierung aufgrund der Herzinsuffizienz, erreichen. Eine geschlechtsspezifische gepoolte Metaanlyse ergab, dass die Effekte von SGLT2 Hemmern auf vaskuläre Risiken und Tod, ebenso wie auf unerwünschte Nebenwirkungen zwischen Männern und Frauen vergleichbar war [66]. Eine geschlechtsspezifische Analyse der TOPCAT-Studie (52 % Frauen) zeigte, dass Frauen im Gegensatz zu Männern über das ganze Spektrum der Herzinsuffizienz von einer Spironolaktontherapie profitieren [67], im amerikanischen Studienarm war die Gesamtmortalität nur bei Frauen signifikant vermindert [68]. In der PARAGON-HF Studie (52 % Frauen) wurde unter Therapie mit ARNI (Sacubitril valsartan) in einer präspezifizierten Subgruppenanalyse nur bei Frauen eine signifikante (−27 %) Reduktion des primären Endpunkts (kardiovaskulärer Tod und Hospitalisierung aufgrund der Herzinsuffizienz) erreicht, auch wenn das Ergebnis der verminderten Hospitalisierungsrate zuzuschreiben war [69, 70]. Die Frauen wiesen öfter Adipositas, weniger oft eine koronare Herzkrankheit und Diabetes (40 vs. 46 %) auf und waren stärker symptomatisch.

Die nichtinvasive Diagnostik der KHK hat bei Frauen eine besonders niedrige Sensitivität und Spezifität, insbesondere die Ergometrie ist wenig aussagekräftig. Oft liegen auch trotz Beschwerden blande Koronarien im Katheter vor (MINOCA), da v. a. bei jüngeren Frauen mikrovaskuläre funktionelle Störungen oder Spasmen überwiegen [71]. Provokationstests und Stresstests können pathologische Veränderungen aufzeigen.

### Mikrovaskuläre Komplikationen

BMI, Alter und höhere Blutzuckerwerte scheinen bei Männern stärkere Prädiktoren für einen Nierenfunktionsverlust darzustellen. Zu beachten ist des Weiteren, dass Frauen mit Diabetes ein besonders hohes Risiko für Harnwegsinfekte haben, welche konsequent behandelt werden müssen.

Bei Retinopathie und Neuropathie sind bisher nur wenige Geschlechtsunterschiede beschrieben (Tab. 2). In der rezent publizierten Maastricht Studie konnte ein höheres Risiko für Nephropathie, Retinopathie und sensorische Neuropathie bei Männer mit T2DM im Vergleich zu gesunden Männern festgestellt werden. Dies war bei Frauen mit T2DM im Vergleich zu gesunden Frauen nicht der Fall [72].

### Tumoren

Diabetes ist mit einem höheren Krebsrisiko verbunden, wobei Übergewicht eine zusätzliche wichtige Rolle spielt. Frauen mit Diabetes haben ein höheres Risiko für Brustkrebs und ein doppelt so hohes Risiko für Endometriumkarzinome, während bei Männern das Risiko für Prostatakarzinom etwas niedriger ist [73]. Außerdem ist bei beiden Geschlechtern das Risiko für Pankreaskarzinome, Darmkrebs und Leberkrebs deutlich erhöht. Frauen mit Diabetes nehmen seltener an Vorsorgeuntersuchungen (Mammographie) teil [74]. Bei beiden Geschlechtern ist auf die Durchführung der allgemein empfohlenen Screeninguntersuchungen unbedingt zu achten.

### Osteoporose

Diabetes ist mit einem höheren Osteoporose- und Frakturrisiko assoziiert, wobei der Knochenstoffwechsel und die Knochenqualität – selbst bei erhaltener Knochenmasse – ungünstig verändert sind. Männer mit Neuropathie scheinen besonders gefährdet [75]. Männer und Frauen mit Diabetes sollen auf ihr individuelles Osteoporoserisiko untersucht werden.

### Depressionen

Diabetes ist häufig mit depressiven Störungen verbunden, welche bei Frauen doppelt so häufig wie bei Männern diagnostiziert werden, aber bei Männern häufig nicht erkannt werden. Auch Angststörungen und kognitive Einschränkungen werden häufiger bei Frauen mit Diabetes mellitus beobachtet [9]. Es soll deshalb bei beiden Geschlechtern regelmäßig auf das Vorliegen einer Depression geprüft werden (siehe Leitlinie psychische Erkrankungen).

## Zusammenfassung

Auch wenn derzeit noch viele Fragen in Bezug auf biologische und psychosoziale geschlechtsspezifische Aspekte in der Entstehung, Prävention und Therapie des Diabetes offen sind und eine wichtige Aufgabe und Herausforderung für zukünftige Forschung darstellen, muss dennoch auf Basis der derzeitigen stetig zunehmenden Erfahrungen und Erkenntnisse bereits eine geschlechtssensible medizinische Betreuung der Patientinnen und Patienten in der Praxis mit Ziel der Personalisierung der medizinischen Betreuung gewährleistet werden. Insbesondere ist auf eine konsequente leitlinienkonforme Therapie modifizierbarer kardiovaskulärer Risikofaktoren bei beiden Geschlechtern zu achten. Dies sollte auch bereits in jüngeren Altersgruppen konsequent umgesetzt werden.

## Interessenkonflikt

A. Kautzky-Willer hat von folgenden Unternehmen, die auch fördernde Mitglieder der ÖDG sind, Forschungsunterstützungen/Honorare erhalten: Eli Lilly, Novo Nordisk, Sanofi-Aventis. M. Leutner hat keinen Interessenkonflikt in Bezug auf diesen Artikel. H. Abrahamian gibt an, dass kein Interessenkonflikt besteht. L. Frühwald gibt an, dass kein Interessenkonflikt besteht. F. Hoppichler gibt an, dass kein Interessenkonflikt besteht. M. Lechleitner hat von folgenden Unternehmen, die auch fördernde Mitglieder der ÖDG sind, Honorare erhalten: AstraZeneca, Eli Lilly, MSD, Novo Nordisk, Novartis, Sanofi-Aventis. J. Harreiter hat keinen Interessenkonflikt in Bezug auf diesen Artikel.

## References

[1] Legato MJ, Gelzer A, Goland R (2006). Gender-specific care of the patient with diabetes: review and recommendations. Gend Med.

[2] International Diabetes Federation (2021). IDF diabetes atlas.

[3] Statistik Austria. Todesursachenstatistik. 2021. http://www.statistik.at/web_de/statistiken/menschen_und_gesellschaft/gesundheit/todesursachen/index.html. Zugegriffen: 24. Febr. 2022.

[4] Vogel B, Acevedo M, Appelman Y (2021). The Lancet women and cardiovascular disease commission: reducing the global burden by 2030. Lancet.

[5] Seshasai SR, Kaptoge S, Emerging Risk Factors Collaboration (2011). Diabetes mellitus, fasting glucose, and risk of cause-specific death. N Engl J Med.

[6] Kautzky-Willer A, Dorner T, Jensby A (2012). Women show a closer association between educational level and hypertension or diabetes mellitus than males: a secondary analysis from the Austrian HIS. BMC Public Health.

[7] Shah B, Tombeau Cost K, Fuller A (2020). Sex and gender differences in childhood obesity: contributing to the research agenda. BMJ Nutr Prev Health.

[8] Koutny F, Weghuber D, Bollow E (2020). Prevalence of prediabetes and type 2 diabetes in children with obesity and increased transaminases in European German-speaking countries. Analysis of the APV initiative. Pediatr Obes.

[9] Kautzky-Willer A, Harreiter J, Pacini G (2016). Sex and gender differences in risk, pathophysiology and complications of type 2 diabetes mellitus. Endocr Rev.

[10] Ferrannini G, De Bacquer D, Vynckier P (2021). Gender differences in screening for glucose perturbations, cardiovascular risk factor management and prognosis in patients with dysglycaemia and coronary artery disease: results from the ESC-EORP EUROASPIRE surveys. Cardiovasc Diabetol.

[11] Kautzky-Willer A, Handisurya A (2009). Metabolic diseases and associated complications: sex and gender matter!. Eur J Clin Invest.

[12] Anderwald C, Gastaldelli A, Tura A (2011). Mechanism and effects of glucose absorption during an oral glucose tolerance test among females and males. J Clin Endocrinol Metab.

[13] Pischon T, Boeing H, Hoffmann K (2008). General and abdominal adiposity and risk of death in Europe. N Engl J Med.

[14] Tamas V, Kempler P (2014). Sexual dysfunction in diabetes. Handb Clin Neurol.

[15] Harreiter J, Kautzky-Willer A (2018). Sex and gender differences in prevention of type 2 diabetes. Front Endocrinol (Lausanne).

[16] Kamenov ZA (2015). A comprehensive review of erectile dysfunction in men with diabetes. Exp Clin Endocrinol Diabetes.

[17] Mazzilli R, Imbrogno N, Elia J (2015). Sexual dysfunction in diabetic women: prevalence and differences in type 1 and type 2 diabetes mellitus. Diabetes Metab Syndr Obes.

[18] Rochester-Eyeguokan C, Meade L (2017). A practical approach to managing hypoactive sexual desire disorder in women with diabetes. Diabetes Ther.

[19] Simon JA, Clayton AH, Kim NN (2022). Clinically meaningful benefit in women with hypoactive sexual desire disorder treated with flibanserin. Sex Med.

[20] Zajecka J (2001). Strategies for the treatment of antidepressant-related sexual dysfunction. J Clin Psychiatry.

[21] Rosen RC, Riley A, Wagner G (1997). The international index of erectile function (IIEF): a multidimensional scale for assessment of erectile dysfunction. Urology.

[22] Wiegel M, Meston C, Rosen R (2005). The female sexual function index (FSFI): cross-validation and development of clinical cutoff scores. J Sex Marital Ther.

[23] Glechner A, Harreiter J, Gartlehner G (2015). Sex-specific differences in diabetes prevention: a systematic review and meta-analysis. Diabetologia.

[24] Gong Q, Zhang P, Wang J (2019). Morbidity and mortality after lifestyle intervention for people with impaired glucose tolerance: 30-year results of the Da Qing Diabetes Prevention Outcome Study. Lancet Diabetes Endocrinol.

[25] Wadden TA, Neiberg RH, Wing RR (2011). Four-year weight losses in the Look AHEAD study: factors associated with long-term success. Obesity (Silver Spring).

[26] Wittert G, Bracken K, Robledo KP (2021). Testosterone treatment to prevent or revert type 2 diabetes in men enrolled in a lifestyle programme (T4DM): a randomised, double-blind, placebo-controlled, 2-year, phase 3b trial. Lancet Diabetes Endocrinol.

[27] Donahue RP, Rejman K, Rafalson LB (2007). Sex differences in endothelial function markers before conversion to pre-diabetes: does the clock start ticking earlier among women? The Western New York Study. Diabetes Care.

[28] Wannamethee SG, Papacosta O, Lawlor DA (2012). Do women exhibit greater differences in established and novel risk factors between diabetes and non-diabetes than men? The British Regional Heart Study and British Women’s Heart Health Study. Diabetologia.

[29] Aroda VR, Christophi CA, Edelstein SL (2015). The effect of lifestyle intervention and metformin on preventing or delaying diabetes among women with and without gestational diabetes: the Diabetes Prevention Program outcomes study 10-year follow-up. J Clin Endocrinol Metab.

[30] Cioana M, Deng J, Nadarajah A (2022). Prevalence of polycystic ovary syndrome in patients with pediatric type 2 diabetes: a systematic review and meta-analysis. JAMA Netw Open.

[31] Kacerovsky-Bielesz G, Lienhardt S, Hagenhofer M (2009). Sex-related psychological effects on metabolic control in type 2 diabetes mellitus. Diabetologia.

[32] Schunk M, Reitmeir P, Schipf S (2012). Health-related quality of life in subjects with and without Type 2 diabetes: pooled analysis of five population-based surveys in Germany. Diabet Med.

[33] Kautzky-Willer A, Kosi L, Lin J (2015). Gender-based differences in glycaemic control and hypoglycaemia prevalence in patients with type 2 diabetes: results from patient-level pooled data of six randomized controlled trials. Diabetes Obes Metab.

[34] Mauvais-Jarvis F, Bairey Merz N, Barnes PJ (2020). Sex and gender: modifiers of health, disease, and medicine. Lancet.

[35] Schütt M, Zimmermann A, Hood R (2015). Gender-specific effects of treatment with lifestyle, metformin or sulfonylurea on glycemic control and body weight: a German multicenter analysis on 9 108 patients. Exp Clin Endocrinol Diabetes.

[36] Kautzky-Willer A, Harreiter J (2017). Sex and gender differences in therapy of type 2 diabetes. Diabetes Res Clin Pract.

[37] Mauvais-Jarvis F, Berthold HK, Campesi I (2021). Sex- and gender-based pharmacological response to drugs. Pharmacol Rev.

[38] Bhattarai M, Salih M, Regmi M (2022). Association of sodium-glucose cotransporter 2 inhibitors with cardiovascular outcomes in patients with type 2 diabetes and other risk factors for cardiovascular disease: a meta-analysis. JAMA Netw Open.

[39] Eberly LA, Yang L, Eneanya ND (2021). Association of race/ethnicity, gender, and socioeconomic status with sodium-glucose cotransporter 2 inhibitor use among patients with diabetes in the US. JAMA Netw Open.

[40] Raparelli V, Elharram M, Moura CS (2020). Sex differences in cardiovascular effectiveness of newer glucose-lowering drugs added to metformin in type 2 diabetes mellitus. J Am Heart Assoc.

[41] Kautzky-Willer A, Kamyar MR, Gerhat D (2010). Sex-specific differences in metabolic control, cardiovascular risk, and interventions in patients with type 2 diabetes mellitus. Gend Med.

[42] Kramer CK, Campbell S, Retnakaran R (2019). Gestational diabetes and the risk of cardiovascular disease in women: a systematic review and meta-analysis. Diabetologia.

[43] Goossens GH, Jocken JWE, Blaak EE (2021). Sexual dimorphism in cardiometabolic health: the role of adipose tissue, muscle and liver. Nat Rev Endocrinol.

[44] Gouni-Berthold I, Berthold HK, Mantzoros CS (2008). Sex disparities in the treatment and control of cardiovascular risk factors in type 2 diabetes. Diabetes Care.

[45] Clemens KK, Woodward M, Neal B (2020). Sex disparities in cardiovascular outcome trials of populations with diabetes: a systematic review and meta-analysis. Diabetes Care.

[46] Mora S, Glynn RJ, Hsia J (2010). Statins for the primary prevention of cardiovascular events in women with elevated high-sensitivity C-reactive protein or dyslipidemia: results from the justification for the use of Statins in prevention: an intervention trial evaluating rosuvastatin (JUPITER) and meta-analysis of women from primary prevention trials. Circulation.

[47] Fulcher J, O’Connell R, Voysey M (2015). Efficacy and safety of LDL-lowering therapy among men and women: meta-analysis of individual data from 174,000 participants in 27 randomised trials. Lancet.

[48] Puri R, Nissen SE, Shao M (2014). Sex-related differences of coronary atherosclerosis regression following maximally intensive statin therapy: insights from SATURN. JACC Cardiovasc Imaging.

[49] King P, Puri R, Ballantyne C (2018). Sex-related difference in the regression of coronary atherosclerosis with the PCSK9 inhibitor, evolocumab: insights from GLAGOV. J Am Coll Cardiol.

[50] d’Emden MC, Jenkins AJ, Li L (2014). Favourable effects of fenofibrate on lipids and cardiovascular disease in women with type 2 diabetes: results from the Fenofibrate Intervention and Event Lowering in Diabetes (FIELD) study. Diabetologia.

[51] Sullivan JC (2008). Sex and the renin-angiotensin system: inequality between the sexes in response to RAS stimulation and inhibition. Am J Physiol Regul Integr Comp Physiol.

[52] Ridker PM, Cook NR, Lee IM (2005). A randomized trial of low-dose aspirin in the primary prevention of cardiovascular disease in women. N Engl J Med.

[53] Gelbenegger G, Postula M, Pecen L (2019). Aspirin for primary prevention of cardiovascular disease: a meta-analysis with a particular focus on subgroups. BMC Med.

[54] Mosca L, Benjamin EJ, Berra K (2011). Effectiveness-based guidelines for the prevention of cardiovascular disease in women—2011 update: a guideline from the american heart association. Circulation.

[55] American Diabetes Association Professional Practice Committee (2021). 10. Cardiovascular disease and risk management: Standards of medical care in diabetes—2022. Diabetes Care.

[56] Bowman L, Mafham M, ASCEND Study Collaborative Group (2018). Effects of aspirin for primary prevention in persons with diabetes mellitus. N Engl J Med.

[57] International Aspirin Foundation. European guidelines aspirin. 2021. https://www.aspirin-foundation.com/scientific-information/guidelines/european-guidelines-aspirin/. Zugegriffen: 4. Mai 2022, updated 27.05.2021.

[58] Regitz-Zagrosek V, Oertelt-Prigione S, EUGenMed Cardiovascular Clinical Study Group (2016). Gender in cardiovascular diseases: impact on clinical manifestations, management, and outcomes. Eur Heart J.

[59] Prospective Studies Collaboration, Asia Pacific Cohort Studies Collaboration (2018). Sex-specific relevance of diabetes to occlusive vascular and other mortality: a collaborative meta-analysis of individual data from 980 793 adults from 68 prospective studies. Lancet Diabetes Endocrinol.

[60] Malmborg M, Schmiegelow MDS, Norgaard CH (2020). Does type 2 diabetes confer higher relative rates of cardiovascular events in women compared with men?. Eur Heart J.

[61] Harreiter J, Fadl H, Kautzky-Willer A (2020). Do women with diabetes need more intensive action for cardiovascular reduction than men with diabetes?. Curr Diab Rep.

[62] Ohkuma T, Komorita Y, Peters SAE (2019). Diabetes as a risk factor for heart failure in women and men: a systematic review and meta-analysis of 47 cohorts including 12 million individuals. Diabetologia.

[63] Fujita Y, Morimoto T, Tokushige A (2022). Women with type 2 diabetes and coronary artery disease have a higher risk of heart failure than men, with a significant gender interaction between heart failure risk and risk factor management: a retrospective registry study. BMJ Open Diabetes Res Care.

[64] Gurgoze MT, van der Galien OP, Limpens MAM (2021). Impact of sex differences in co-morbidities and medication adherence on outcome in 25 776 heart failure patients. ESC Heart Fail.

[65] Gerdts E, Regitz-Zagrosek V (2019). Sex differences in cardiometabolic disorders. Nat Med.

[66] Radholm K, Zhou Z, Clemens K (2020). Effects of sodium-glucose co-transporter-2 inhibitors in type 2 diabetes in women versus men. Diabetes Obes Metab.

[67] Solomon SD, Claggett B, Lewis EF (2016). Influence of ejection fraction on outcomes and efficacy of spironolactone in patients with heart failure with preserved ejection fraction. Eur Heart J.

[68] Merrill M, Sweitzer NK, Lindenfeld J (2019). Sex differences in outcomes and responses to spironolactone in heart failure with preserved ejection fraction: a secondary analysis of TOPCAT trial. JACC Heart Fail.

[69] Solomon SD, McMurray JJV, Anand IS (2019). Angiotensin-Neprilysin inhibition in heart failure with preserved ejection fraction. N Engl J Med.

[70] McMurray JJV, Jackson AM, Lam CSP (2020). Effects of Sacubitril-Valsartan versus Valsartan in women compared with men with heart failure and preserved ejection fraction: insights from PARAGON-HF. Circulation.

[71] Pacheco Claudio C, Quesada O, Pepine CJ (2018). Why names matter for women: MINOCA/INOCA (myocardial infarction/ischemia and no obstructive coronary artery disease). Clin Cardiol.

[72] de Ritter R, Sep SJS, van der Kallen CJH (2021). Sex differences in the association of prediabetes and type 2 diabetes with microvascular complications and function: The Maastricht Study. Cardiovasc Diabetol.

[73] Clayton PE, Banerjee I, Murray PG (2011). Growth hormone, the insulin-like growth factor axis, insulin and cancer risk. Nat Rev Endocrinol.

[74] Lipscombe LL, Hux JE, Booth GL (2005). Reduced screening mammography among women with diabetes. Arch Intern Med.

[75] Rasul S, Ilhan A, Wagner L (2012). Diabetic polyneuropathy relates to bone metabolism and markers of bone turnover in elderly patients with type 2 diabetes: greater effects in male patients. Gend Med.

[76] Stolarz AJ, Rusch NJ (2015). Gender differences in cardiovascular drugs. Cardiovasc Drugs Ther.

[77] Harreiter J, Kautzky-Willer A (2017). Mann oder Frau: Ist das bei Diabetes relevant?. MMW Fortschr Med.

